# Primary tuberculosis of the glans penis: A rare presentation and review of literature

**DOI:** 10.1016/j.eucr.2021.101858

**Published:** 2021-09-20

**Authors:** Abdoul Kader Tapsoba, Mokhtar Bibi, Tiéoulé Mamadou Traoré, Asma Ayari, Sami Ben Rhouma, Yassine Nouira

**Affiliations:** a University of Joseph Ki-Zerbo, Ouagadougou, Burkina Faso; b Departement of Urology, La Rabta Hospital, Tunis, 1001, Tunisia; c University Tunis Manar, Faculty of Medicine of Tunis, Department of Urology, La Rabta Hospital, Tunis, Tunisia; d University of Ouahigouya, Ouahigouya, Burkina Faso; e University Tunis Manar, Faculty of Medicine Tunis, Department of Pathology, La Rabta Hospital, Tunis, 1001, Tunisia

**Keywords:** Glans tuberculosis, Ulcerative lesions, Antitubercular treatement

## Abstract

Tuberculosis poses a real public health problem inTunisia.We report an 80-year old patient, immunocompetent, who has initial presentation of a painless ulcerative lesions of the glans penis. A history of pulmonary tuberculosis was not present.The physical examination revealed an indurated glans penis covered with blackish crusts and small ulcerations. A biopsy of the lesion was undertaken and histological examination was confirmed the diagnosis of tuberculosis of glans.Others investigations was performed without finding any abnormalities He responded well to anti-tubercular treatment with complete resolution of lesions in six months.

## Highlights

• Tuberculosis poses a real public health problem in endemic countries.
• Tuberculosis of the glans penis is an uncommon presentation of tuberculosis.This is the first case to be reported in Africa.
• Clinical presentation is extremely varied.
• Diagnostic confirmation is based on histological study aided by culture.
• Antibiotic therapy for tuberculosis is the basis of treatment.

## 1 Introduction

Tuberculosis(TB) poses a real public health problem in Tunisia and in developing countries**.** Globally, no country has ever been able to eradicate TB.The cutaneous form represents 2% of all of extra-pulmonary tuberculosis and localization on the glans is even rarer.^1^ TB of the glans penis is an uncommon presentation of TB, even in countries where the incidence of pulmonary and extrapulmonary TB is high.

We report an 80-year old patient, immunocompetent,who was referred to our institution for multiple ulcerative necrotic lesions of the glans penis.A biopsy of the lesion was undertaken and histological examination confirmed the diagnosis of tuberculosis of glans.Clinical and pathological features will be discussed with a literature review of the previous cases.

## 2 Presentation of case

An 80-year-old Tunisian man was referred to our institution for multiple ulcerative necrotic lesions of the glans penis that had been evolving for 15 days, suggestive of necrotizing dermatitis.the patient would have noticed the appearance of a small punctiform ulceration on the anterior face of the glans penis that progressively increased in size, taking up almost the entire glans penis.

He has no history of pulmonary tuberculosis, he had an endoscopic resection of the prostate in 2000 for benign prostatic hyperplasia, he is married and denies having had other sexual partners in the past months.

**Fig. 1:**
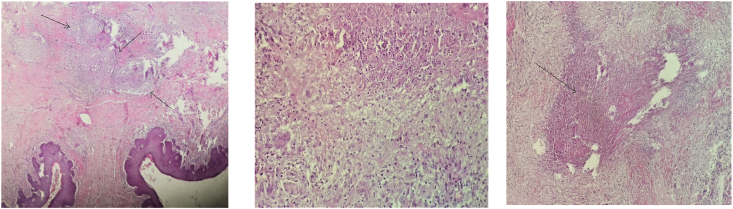
(a)confluent epithelioid granulomas (Haematoxylin and eosin x10). (b)Epithelioid granuloma with giant cells (HEX20). (c) Caseous necrosis (HEX20).

The physical examination revealed an indurated glans penis covered with blackish crusts and small ulcerations of 0.5 cm in diameter and 0.5 cm in depth, and the glans penis was painless on palpation. He had no inguinal nodes. The rest of the genital examination was normal. HIV serology was negative. Excision of the lesions was then undertaken and histological examination of the specimen showed confluent epithelioid granulomas, an epithelioid granuloma with giant cells and caseous necrosis [Fig. 1 (a,b,c)]. All microbiological staining of the specimen was negative. Culture of the collected tissues revealed Mycobacterium tuberculosis, susceptible to isoniazid, rifampicin, pyrazinamide, streptomycin and ethambutol.

Early morning urine samples were negative for mycobacteria on microscopy and culture. Cytological tests were also negative. Intravenous urography did not reveal any abnormalities in other structures of the urinary tract.

He received anti-tuberculosis treatment for 6 months and we noted a good evolution of the lesions.The patient was cured without any recurrence after 15 months of follow-up.

## 3 Discussion

Tuberculosis is still a major cause of morbidity in developing countries like Tunisia. An extremely rare form of genitourinary tract tuberculosis will present as tuberculosis of glans penis constituting less than 1% of reported cases worldwide. Epididymis (42%) followed by seminal vesicle (23%), prostrate (21%), testis (15%) and vas deferens (12%) are the common sites of presentation.^2^

Based on a systematic Pubmed search using the keywords « tuberculosis of glans penis», we have not found many cases published in the literature. We have excluded those who could not be opened. Most of the published cases are case reports.

Fournier was stated to have described in 1848 the first case of a patient with TB of penis who had multiple ulcers of the penis and regional lymphadenopath.Lewis in 1946 reviewed 110 cases of TB of the penis and Sekhon in 1971 reviewed 29 cases of TB of penis that had been reported between 1946 and 1971. From 1971 through 1999, further 16 cases of TB of the penis had been reported by a number of authors in the literature(3).Since 2000 to 2020 fifteen cases of TB of the glans penis have been sporadically reported (Table 1).

**Table 1:** Summary of the cases of tuberculosis of glans penis.

**Autors**	**Years**	**Nombers of patients**	**symptoms**	**Country**	Histological findings	**Treatement**	Outcome
Brian J.Angus	2001	01	painless ulcer on penis	United kingdom	caseating granuloma and tissue culture of the biopsy yielded Mycobacterium tuberculosis.	ATT	Would healed well with no recurrence.
Amir-zargar ma	2004	01	An ulcerative burgeon (granulated) lesion on his glans	Iran	section biopsy was performed and the pri-mary report showed tuberculosis	ATT	**Complete improvement was achieved following the treatment**
Shigenori Yonemura	2004	01	painless subcutaneous nodule at the glans penis	Japan	granulomatous inflammation. Tuberculin tests were strongly positive,	ATT	**complete healing occurred**
Baveja	2007	01	multiple ulcers on glans penis;	India	Direct smear microscopy of pus showed heavy growth of acid fast bacillus (3+); ELISA serology for mycobacterium A60-antigen was strongly positive.	ATT	**Ulcer healed after 3 months**
Ghorbani	2007	01	painful ulcer on glans penis.	Iran	Biopsy of the ulcer and PCR revealed Mycobacterium tuberculosis.	ATT	**Ulcer healed.**
KishanChand	2009	01	ulcer on glans penis with everted edges distorting the external urethral meatus; tender bilateral inguinal lymph node enlargement.	India	chronic granuloma	ATT	**Ulcer heale**
J. K. Kar	2012	01	ulcer on glans penis	India	Biopsy of ulcerated lesion histology showed TB of penis	ATT	**Responded well and ulcer healed**
Jose David Jimenez Parra	2014	01	papular-ampullary eruption in the glans penis	Espagne	a chronic granulomatous inflammatory necrotizing lesion with granulomatous vasculitis lesions, without tumor infiltration	ATT	**five months the ulceration healed significantly.**
Deb S	2015	01	multiple ulcers over the glans penis and around the urethral meatus.	India	Numerous Langhans type of giant cells were seen with lymphoid cell infiltration	ATT	**complete subsidence of the lesions after 6 months**
Khan D	2016	01	ulceration over the glans penis for3 month	India	Histopathology revealedlymphoplasmacytic infiltration of subepithelial fibrocollage-nous tissue along with the presence of epithelioid granulomawith caseation, suggesting a mycobacterial infection (	ATT	**after 6 months it healedcompletely.**
Gangalakshmi C, Sankaramahalingam	2016	01	ulcerative growth over glans penis	India	epithelioid cell granuloma and Langhan's giant cells suggestive of tuberculoid granuloma	ATT	**Within 3 weeks, patient showed signs of healing**
Archana Singal	2017	01	multiple ulcers on the glans penis	India	Histopathology revealed the diagnosis of genital tuberculosis (TB), and polymerase chain reaction for Mycobacterium tuberculosis tested positive	ATT	**complete resolution of lesions in six months**
D Pandey	2019	01	multiple undermined ulcers	Nepal	histopathology revealed a diagnosis of tuberculous ulcer	ATT	**responded well to antitubercular therapy**
John S Banerji	2020	01	painful ulcerative lesion of the glans penis	USA		ATT	**completely healed glans penis lesion after antituberculosis therapy**
Y.Elkhachine	2020	01	Chronic ulcerative lesions on glans penis	Morocco	A skin biopsy, performed at the site of an ulceration, showed the presence of epithelial-giganto-cellular granulomas with caseous necrosis in favor of a cutaneous tuberculosis	ATT	**Ulcer healed**

ATT:antitubercular treatement.

Clinical presentation is extremely varied, and TB of the penis may present as painless nodules, indurated swelling of the glans, single or multiple genital ulcers, fungating growth and urethral discharge with or without erectile dysfunction.^4^

TB of the glans penis may either be primary TB or secondary TB but on the whole most of the reported cases have been primary TB.^3^ Primary penile tuberculosis are usually acquired either through violent intercourse with female partners with active genital tuberculosis or infected patients own ejaculates or through fomite spread by contact with infected cloths.^2^ The secondary form is due to the subsequent complication of lung tuberculosis or TB of other parts of urogenital tract extended through urethra or through haematogenous route.

Diagnostic confirmation is based on histological study aided by culture, which represents the gold standard but which requires six to eight weeks for the isolation of BK. PCR allows an equivalent result in 24–48 hours.^5^

The diagnosis requires the search for other tuberculous sites, in particular an attack on the urinary tract, by performing an intravenous urography, a direct bacteriological examination.These examinations were normal in our patient. Similarly, the HIV infection is essential because of the frequency and increased morbidity and mortality of tuberculosis in this context.Our patient's HIV serology was negative. Examination of the partner is indicated for urogenital tuberculosis.The absence of another tuberculous localization, notably urogenital, in our patient eliminates a secondary tuberculosis in particular orificial tuberculosis.

Antibiotic therapy for tuberculosis is the basis of treatment.It allowed a rapid healing of the ulcerations in our patient, confirming a posteriori the diagnosis.

## 4 Conclusion

Tuberculosis should be considered in the presence of ulceration of the glans, particularly in countries with a high endemic.Skin biopsy should be performed systematic to eliminate a malignant tumor of the penis. Determine whether a TB of glans penis is a primary or a secondary disease, intravenous pyelography and chest X-ray must be done. Antitubercular drugs are the mainstay of treatment.

## Consent

Written informed consent was obtained from the patient for publication of this case report and accompanying images.

## Funding source

His research did not receive any specific grant from funding agencies in the public, commercial, or not-

## Author contribution

All authors have contributed to this work and have read and approved the final version of the manuscript.

## Declaration of competing interest

The authors declare that they have no conflicts of interest.
